# Effect of the Duration of NSAID Use on COVID-19

**DOI:** 10.3390/medicina58121713

**Published:** 2022-11-23

**Authors:** Kyeongmi Kim, Siyeoung Yoon, Junwon Choi, Soonchul Lee

**Affiliations:** 1 Department of Laboratory Medicine, CHA Ilsan Medical Center, School of Medicine, CHA University, 100 Ilsan-ro, Ilsandong-gu, Goyang-si 10444, Republic of Korea; 2 Department of Orthopaedic Surgery, CHA Bundang Medical Center, School of Medicine, CHA University, 335 Pangyo-ro, Bundang-gu, Seongnam-si 13488, Republic of Korea; 3 Department of Molecular Science and Technology, Department of Applied Chemistry and Biological Engineering, Ajou University, 206 World cup-ro, Yeongtong-gu, Suwon-si 16499, Republic of Korea

**Keywords:** COVID-19, NSAID, duration

## Abstract

*Background and Objectives:* Non-steroidal anti-inflammatory drugs (NSAIDs) are commonly used to control pain and fever. However, their effect on COVID-19 infected patients has not been fully studied. In this study, we investigated the effect of the duration of NSAIDs use on COVID-19 infection and clinical outcomes. *Materials and Methods:* In South Korea, 25,739 eligible patients who received COVID-19 testing between 1 January and 31 July 2020, were included in this retrospective observational cohort analysis. Based on the date of the first COVID-19 test for each patient, NSAID prescription dates were used to separate patients into two groups (short-term group: <2 weeks; long-term group: 8–12 weeks). COVID-19 infectivity and clinical outcomes were analyzed. We used the propensity score-matching (PSM) method. *Results:* Of the 580 patients who had taken NSAIDs before the date of COVID-19 test, 534 and 46 patients were grouped in the short- and long-term NSAID-use groups, respectively. We did not find a statistically significant increased risk of COVID-19 infection (adjustment for age and sex, *p* = 0.413; adjustment for age, sex, region of residence, comorbidity, Charlson Comorbidity Index, and current use of medication, *p* = 0.259) or change in clinical outcomes, including conventional oxygen therapy, admission of intensive care unit, artificial ventilation, or death, between the two groups in which the PSM method was applied. *Conclusions:* The duration of NSAIDs use did not have a statistically significant effect on COVID-19 infectivity or clinical outcomes. However, further studies looking at clinical presentation and laboratory test results in a large number of people should be performed.

## 1. Introduction

In December 2019, the Coronavirus disease 2019 (COVID-19), caused by a novel coronavirus (SARS-CoV-2) that causes viral pneumonia, was first discovered in China [1]. It has rapidly spread globally through close human interactions or the respiratory secretions of infected people. On 11 March 2020, the World Health Organization (WHO) has declared the COVID-19 outbreak as a “pandemic” [2]. The number of infected cases and deaths due to COVID-19 is rapidly increasing, and in August 2022, there were more than 58 hundred million confirmed cases, with over 6,400,000 deaths globally [3]. In Korea, six outbreaks have been recorded, with more than 20 million confirmed cases and over 25,332 deaths [4]. The spread of COVID-19 is affecting economic, social, and racial issues, not only in Korea but also around the world.

In COVID-19-infected patients, non-steroidal anti-inflammatory drugs (NSAIDs) such as ibuprofen, along with acetaminophen, are commonly used to control symptoms such as fever and muscle pain. However, French authorities have warned about the risks of using ibuprofen for COVID-19 infection, and there are reports of serious COVID-19 cases requiring intensive care after exposure to ibuprofen in young patients [5,6]. Our previous study reported that aspirin, a class of NSAIDs, had a negative effect on clinical outcomes in patients with COVID-19 infection [7]. However, according to a meta-analysis by Moore et al., NSAIDs use does not increase the risk of COVID-19 infection, hospital admission, severe COVID-19, or death [8]. In addition, studies on the length of NSAIDs exposure revealed that NSAIDs had no effect on the infection and severity of COVID-19 with various exposure periods, including current or acute exposure [9,10] or within 30 days [11] or 4 months [12]. However, NSAIDs use in COVID-19 patients is still controversial, and there is a lack of sufficient research on the effect of NSAIDs on COVID-19 depending on the duration of use.

This study aimed to evaluate the difference in COVID-19 infection and clinical outcomes according to the duration of NSAIDs use. We analyzed patients by subdividing them according to the duration of NSAID use before the diagnosis of COVID-19 using nationwide Korean COVID-19 data.

## 2. Materials and Methods

### 2.1. Data Source

We analyzed the database of Korean National Health Insurance System (NHIS)-COVID-19 cohort, which includes data on all patients tested for COVID-19 in South Korea from 1 January to 31 July 2020 (*N* = 25,739). The COVID-19 testing date, results, and demographic information were all included in the data. Furthermore, the database contains codes for disease diagnosis, prescription information, outcomes related to COVID-19, and death records. All of the personal information used in this study was de-identified.

### 2.2. Study Population

The cohort’s index date was the first COVID-19 test for each patient. We included all patients aged 20 years and older with a history of taking NSAIDs among patients who tested positive for COVID-19 between 1 January and 31 July 2020 (*N* = 580). We extracted and combined patient characteristics such as sex, age (20–29, 30–39, 40–49, 50–59, 60–69, 70–79, 80+), and region of residence (Seoul, Gyeonggi, Daegu, Gyeongbuk, etc.). At least two claims were evaluated within a year to confirm the presence of underlying diseases (such as asthma, cerebrovascular disease (CVD), chronic kidney disease (CKD), chronic obstructive pulmonary disease (COPD), diabetes mellitus (DM), hypertension (HTN)) and the Charlson comorbidity index score was calculated using previously reported methods [13]. Systemic steroid use within 30 days of the index date was analyzed using the types and codes obtained from a previous study [14].

### 2.3. Study Group Classification

We used NHIS-COVID-19 database’s inpatient and outpatient prescription records for NSAIDs including both oral and intravenous formulations. We classified 580 patients prescribed NSAIDs according to treatment duration. From the index data, patients who took NSAIDs within 2 weeks were classified into the short-term group, and those who had taken NSAIDs for more than 8 weeks and within 12 weeks were classified into the long-term group.

### 2.4. Outcomes

The outcomes included composite endpoint 1 and composite endpoint 2. Composite end point 1 contains conventional oxygen therapy, admission of intensive care unit (ICU), artificial ventilation, or death. Composite end point 2 contains admission of ICU, artificial ventilation, or death except for conventional oxygen therapy in composite end point 1.

### 2.5. Statistis

*p*-values less than 0.05 were considered significant. Pearson’s χ2 test or Fisher’s exact test used data analysis. To reduce potential bias caused by differences in patients’ baseline characteristics, we used the propensity score matching (PSM) method. A logistic regression model was used, with adjustments for the variables. We calculated the PSM of the two groups at a 1:1 fixed ratio based on a greedy nearest neighbor algorithm and estimated the predicted probability of short-term versus long-term NSAIDs users. We examined standardized mean differences (SMDs) across groups to detect any remaining imbalance in the matched samples [15]. Data processing and statistical analysis were performed using R software, version 3.6.3 (The R Foundation for Statistical Computing, Vienna, Austria).

## 3. Results

### 3.1. Study Subjects

This retrospective analysis was performed on patients enrolled in the NHIS-COVID-19 cohort database due to a positive COVID-19 result via real-time reverse transcription polymerase chain reaction (RT-PCR). Of the 25,739 individuals who tested positive for COVID-19, 580 had taken NSAIDs prior to the positive result. Among them, 534 patients (92.07%) used NSAIDs within 2 weeks and 46 (7.93%) patients used NSAIDs for more than 8 weeks and within 12 weeks before the index date.

### 3.2. Baseline Characteristics

The baseline patient characteristics are shown in Table 1. Among NSAIDs users, 271 (53.3%) were women and 309 (46.7%) were men. In the short-term group, the age group with the most participants (20.4%) was the 50–59 age group, whereas in the long-term group, the 60–69 age group and 70–79 age group were the most common. Hypertension was the most common comorbidity observed in the two groups. In the long-term group, COPD, DM, and hypertension were also significantly higher than those in the short-term group, but there was no difference in asthma, CKD, and CVD. The Charlson comorbidity index score was significantly higher in the long-term group (Table 1).

### 3.3. Propensity Score-Matching

Short- and long-term NSAIDs users before COVID-19 diagnosis were individually matched to an equal number of long-term NSAIDs use patients in our PSM cohorts (Table 2). When SMD was used to analyze the demographics and clinical variables in the PSM cohorts, no significant differences were found in either (SMD < 0.25). In the long-term group, the minimally adjusted odds accounting for age and gender via PSM of COVID-19 positivity were 1.5 (95% CI, 0.57–4.05), and the fully adjusted odds accounting for all variables included in this analysis were 2.24 (95% CI, 0.56–9.57). Patients who used NSAIDs for a long time were more likely to be confirmed to have COVID-19, but this was not statistically significant (minimally adjusted, *p* = 0.413; fully adjusted, *p* = 0.259).

### 3.4. Clinical Outcome

In Table 3, the endpoints of 13 patients in the short-term group and 17 patients in the long-term group with confirmed clinical outcomes were analyzed. The composite endpoints 1 and 2 were 15.4% (2/13) and 7.7% (1/13) in the short-term group and 47.1% (8/17) and 23.5% (4/17) in the long-term group, respectively. Patients in the long-term group had a higher probability of experiencing severe clinical outcomes, but this was not statistically significant (composite endpoint 1, *p* = 0.119; composite endpoint 2, *p* = 0.355).

## 4. Discussion

NSAIDs are among the most-prescribed drugs worldwide, constituting 5% of all prescribed drugs [16]. NSAIDs are mostly used to relieve pain, reduce inflammation, and decrease fever, and even used extensively as analgesics [16,17]. NSAIDs exhibit anti-inflammatory properties by decreasing the biosynthesis of prostaglandin (PG) through the inhibition of intracellular cyclo-oxygenase enzymes [18]. NSAIDs are indicated for rheumatoid arthritis, osteoarthritis, fever, headache, and dysmenorrhea and have been shown to prevent Alzheimer’s dementia [19]. However, despite the many indications mentioned above, NSAIDs should be used with caution as they may have adverse effects on a variety of organs, including the respiratory tract [19,20].

French officials warned against using ibuprofen, one of the NSAIDs, in patients with COVID-19 symptoms in March 2020 [6]. Initially, the European Medicines Agency, Medicines and Healthcare Products Regulatory Agency in the UK, and the WHO recommended against using ibuprofen [21,22,23]. Similarly, the Korea Centers for Disease Control and Prevention (KCDC) recommended the use of acetaminophen instead of NSAIDs for symptom relief [24]. The cell entry receptor for SARS-CoV-2 is angiotensin-converting enzyme 2 (ACE2), and previous research has indicated that NSAIDs upregulate this enzyme, thus raising concerns that NSAIDs may increase vulnerability to infection [25,26]. As such, a lower death rate has been reported in patients using ACE inhibitors [26]. There is also a report that NSAIDs use increases the risk of hospital death, ICU admission, artificial ventilation, and sepsis in Korean patients with COVID-19, so that NSAIDs use should be cautioned in COVID-19 patients [27]. Based on the results of these reports, the use of NSAIDs in COVID-19 patients has been questioned in many countries.

However, many studies related to COVID-19 have been conducted, and many have reported that the use of NSAIDs has little effect or positive effects on COVID-19 infection. Chen et al. reported that suppressing COX-2/PGE2 signaling with ibuprofen and meloxicam, two commonly used NSAIDs, had no influence on ACE2 expression, viral entrance, or viral replication [25]. Multi-center studies found that the use of NSAIDs was not correlated with COVID-19. On the contrary, it serves to reduce both the prevalence and severity of COVID-19 infection [10,28]. In addition, exposure to ibuprofen has been reported to be related to a lower risk of hospitalization and artificial ventilation [29]. A meta-analysis of 266 papers mentioning NSAIDs did not confirm that the use of NSAIDs, including ibuprofen, increase the risk of COVID-19 infection, hospital admission, severe COVID-19, or death [8]. Nevertheless, many clinicians and patients are still hesitant to use NSAIDs.

Although the administration of NSAIDs to COVID-19 patients is still controversial, recently published papers support the use of NSAIDs [8,10,28,29]. Accordingly, we attempted to investigate the effect of NSAIDs on COVID-19 using a nationwide Korean cohort to determine whether the duration of NSAIDs use reduced susceptibility to COVID-19. The NHIS-COVID-19 cohort database was created by the Health Insurance Review and Assessment of Korea (HIRA) and the KCDC for use in medical research. We did not observe a statistically significantly higher risk of SARS-CoV-2 infection in the propensity score-matched population with short- and long-term NSAID exposure, which is consistent with the findings of prior research. Furthermore, we found no statistically significant increased risk associated with conventional oxygen therapy, ICU admission, artificial ventilation, or death from COVID-19 between the two groups. Based on several other studies and our analysis, there is currently little scientific data to support the use of NSAIDs increasing the infection rate and severity of clinical outcomes with COVID-19.

However, this study had several limitations. First, many patients who were taking long-term NSAIDs were not included in the analysis (7.9% of 580 patients). Furthermore, the number of patients who had clinically confirmed outcomes was minimal (5.2% of 580 patients). Second, because a comparison with the patient group who did not use NSAIDs was not made, it was not possible to analyze whether the use of NSAIDs affected COVID-19 and not only the period of use. Third, unmeasured factors may have influenced the results, despite our best efforts to account for all confounding factors using PSM analysis. Additionally, clinical presentation, symptoms, clinical course, and laboratory test results could not be included because this study was retrospective and used information from the Korean NHIS. Fourth, even though NSAIDs are over-the-counter medicines in Korea, we only analyzed the data for prescribed NSAIDs use.

Regardless of these drawbacks, the study’s information on the effects of NSAID use in Korean COVID-19 patients is reliable. In addition, it is meaningful in that it analyzes how the period of taking NSAIDs affects COVID-19 in Koreans using a large amount of Korean NHIS data, and we used PSM to eliminate the effects of factors other than NSAIDs. However, more large-scale studies of the association between COVID-19 and NSAIDs use are needed, with better control of variables except NSAIDs.

## 5. Conclusions

The duration of NSAIDs use did not have a statistically significant effect on COVID-19 infectivity or severity of clinical outcomes. Therefore, our study results are expected to help physicians and patients decide whether to take NSAIDs in relation to COVID-19 infection. However, further studies focusing on clinical presentation and laboratory test results in a large number of people should be performed.

## Figures and Tables

**Table 1:** Baseline characteristics of patients who were used non-steroidal anti-inflammatory drugs (NSAIDs) for short (2 weeks) or long term (8–12 weeks) duration before the COVID-19 index date in the Korean National Health Insurance System (NHIS) COVID-19 database.

Characteristic	Entire Cohort *N* = 580	Short-Term *N* = 534	Long-Term *N* = 46	*p*-Value
Sex, *n* (%)				0.123
Male	271 (46.7)	244 (45.7)	27 (58.7)	
Female	309 (53.3)	290 (54.3)	19 (41.3)	
Age, *n* (%)				0.009
20–29	79 (13.6)	78 (14.6)	1 (2.2)	
30–39	76 (13.1)	73 (13.7)	3 (6.5)	
40–49	80 (13.8)	76 (14.2)	4 (8.7)	
50–59	118 (20.3)	109 (20.4)	9 (19.6)	
60–69	101 (17.4)	91 (17.0)	10 (21.7)	
70–79	71 (12.2)	61 (11.4)	10 (21.7)	
80+	55 (9.5)	46 (8.6)	9 (19.6)	
Region, *n* (%)				0.171
Seoul	79 (13.6)	75 (14.0)	4 (8.7)	
Gyeonggi	69 (11.9)	67 (12.5)	2 (4.3)	
Daegu	163 (28.1)	144 (27.0)	19 (41.3)	
Gyeongbuk	54 (9.3)	50 (9.4)	4 (8.7)	
Other	215 (37.1)	198 (37.1)	17 (37.0)	
Comorbidity, *n* (%)				
Asthma	102 (17.6)	95 (17.8)	7 (15.7)	0.812
CVA	68 (11.7)	58 (10.9)	10 (21.7)	0.05
CKD	32 (5.5)	28 (5.2)	4 (8.7)	0.517
**COPD**	**40 (6.9)**	**33 (6.2)**	**7 (15.2)**	**0.044**
**DM**	**134 (23.1)**	**113 (21.2)**	**21 (45.7)**	**<0.001**
**HTN**	**184 (31.7)**	**159 (29.8)**	**25 (54.3)**	**0.001**
**Charlson Comorbidity Index, *n* (%)**				**0.032**
0	217 (37.4)	207 (38.8)	10 (21.7)	
1	89 (15.3)	83 (15.5)	6 (13.0)	
2 or more	274 (47.2)	244 (45.7)	30 (65.2)	
Current use of medication, *n* (%)				
Steroid	118 (20.3)	103 (19.3)	15 (32.6)	0.05

**Table 2:** Propensity score-matched baseline characteristics and positive COVID-19 test result between the short- and long-term NSAIDs-use groups.

Characteristic	Short-Term *N* = 46	Long-Term *N* = 46	SMD
Sex, *n* (%)			0.044
Male	26 (56.5)	27 (58.7)	
Female	20 (43.5)	19 (41.3)	
Age, *n* (%)			0.068
20–29	2 (4.3)	1 (2.2)	
30–39	3 (6.5)	3 (6.5)	
40–49	3 (6.5)	4 (8.7)	
50–59	11 (23.9)	9 (19.6)	
60–69	8 (17.4)	10 (21.7)	
70–79	11 (23.9)	10 (21.7)	
80+	8 (17.4)	9 (19.6)	
Region, *n* (%)			0.213
Seoul	10 (21.7)	4 (8.7)	
Gyeonggi	18 (39.1)	2 (4.3)	
Daegu	1 (2.2)	19 (41.3)	
Gyeongbuk	3 (6.5)	4 (8.7)	
Other	14 (30.4)	17 (37.0)	
Comorbidity, *n* (%)			
Asthma	6 (13.0)	7 (15.7)	0.060
**CVA**	**10 (21.7)**	**10 (21.7)**	**0.000**
CKD	1 (2.2)	4 (8.7)	0.230
COPD	6 (13.0)	7 (15.2)	0.060
**DM**	**20 (43.5)**	**21 (45.7)**	**0.043**
HTN	20 (43.5)	25 (54.3)	0.216
Charlson Comorbidity Index, *n* (%)			0.104
0	8 (17.4)	10 (21.7)	
1	6 (13.0)	6 (13.0)	
2 or more	32 (69.6)	30 (65.2)	
Current use of medication, *n* (%)			
Steroid	18 (39.1)	15 (32.6)	0.138
COVID-19, *n* (%)			
Minimally adjusted OR *	1.00 (reference)	1.5 (0.57–4.05)	
Fully adjusted OR #	1.00 (reference)	2.24 (0.56–9.57)	

* Adjustment for age and sex (*p*-value: 0.413); # Adjustment for age, sex, region of residence, comorbidity, Charlson Comorbidity Index, and current use of medication (*p*-value: 0.259).

**Table 3:** Clinical outcomes of COVID-19-infected patients between the short- and long-term NSAIDs-use groups.

Variables	Short-Term *N* = 13	Long-Term *N* = 17	*p*-Value *
Composite endpoint1, *n* (%)			0.119
No	11 (84.6)	9 (52.9)	
Yes	2 (15.4)	8 (47.1)	
Composite endpoint2, *n* (%)			0.355
No	12 (92.3)	13 (76.5)	
Yes	1 (7.7)	4 (23.5)	

* Fisher’s exact test; Composite endpoint 1: conventional oxygen therapy, admission of intensive care unit (ICU), artificial ventilation, or death. Composite endpoint 2: admission of ICU, artificial ventilation, or Death.

## Data Availability

Data are contained within the article.
